# Signed Distance Correlation (SiDCo): an online implementation of distance correlation and partial distance correlation for data-driven network analysis

**DOI:** 10.1093/bioinformatics/btad210

**Published:** 2023-05-03

**Authors:** Francesco Monti, David Stewart, Anuradha Surendra, Irina Alecu, Thao Nguyen-Tran, Steffany A L Bennett, Miroslava Čuperlović-Culf

**Affiliations:** National Research Council of Canada, Digital Technologies Research Centre, Ottawa, Ontario, Canada; Neural Regeneration Laboratory and India Taylor Lipidomic Research Platform, Ottawa, Ontario, Canada; National Research Council of Canada, Digital Technologies Research Centre, Ottawa, Ontario, Canada; Neural Regeneration Laboratory and India Taylor Lipidomic Research Platform, Ottawa, Ontario, Canada; National Research Council of Canada, Digital Technologies Research Centre, Ottawa, Ontario, Canada; Neural Regeneration Laboratory and India Taylor Lipidomic Research Platform, Ottawa, Ontario, Canada; Neural Regeneration Laboratory and India Taylor Lipidomic Research Platform, Ottawa, Ontario, Canada; Department of Biochemistry, Microbiology, and Immunology and Ottawa Institute of Systems Biology, Ottawa, Ontario, Canada; Neural Regeneration Laboratory and India Taylor Lipidomic Research Platform, Ottawa, Ontario, Canada; Department of Biochemistry, Microbiology, and Immunology and Ottawa Institute of Systems Biology, Ottawa, Ontario, Canada; Department of Chemistry and Biomolecular Sciences, Centre for Catalysis Research and Innovation, University of Ottawa, Ottawa, Ontario, Canada; Neural Regeneration Laboratory and India Taylor Lipidomic Research Platform, Ottawa, Ontario, Canada; Department of Biochemistry, Microbiology, and Immunology and Ottawa Institute of Systems Biology, Ottawa, Ontario, Canada; Department of Chemistry and Biomolecular Sciences, Centre for Catalysis Research and Innovation, University of Ottawa, Ottawa, Ontario, Canada; National Research Council of Canada, Digital Technologies Research Centre, Ottawa, Ontario, Canada; Neural Regeneration Laboratory and India Taylor Lipidomic Research Platform, Ottawa, Ontario, Canada; Department of Biochemistry, Microbiology, and Immunology and Ottawa Institute of Systems Biology, Ottawa, Ontario, Canada

## Abstract

**Motivation:**

There is a need for easily accessible implementations that measure the strength of both linear and non-linear relationships between metabolites in biological systems as an approach for data-driven network development. While multiple tools implement linear Pearson and Spearman methods, there are no such tools that assess distance correlation.

**Results:**

We present here SIgned Distance COrrelation (SiDCo). SiDCo is a GUI platform for calculation of distance correlation in omics data, measuring linear and non-linear dependencies between variables, as well as correlation between vectors of different lengths, e.g. different sample sizes. By combining the sign of the overall trend from Pearson’s correlation with distance correlation values, we further provide a novel “signed distance correlation” of particular use in metabolomic and lipidomic analyses. Distance correlations can be selected as one-to-one or one-to-all correlations, showing relationships between each feature and all other features one at a time or in combination. Additionally, we implement “partial distance correlation,” calculated using the Gaussian Graphical model approach adapted to distance covariance. Our platform provides an easy-to-use software implementation that can be applied to the investigation of any dataset.

**Availability and implementation:**

The SiDCo software application is freely available at https://complimet.ca/sidco. Supplementary help pages are provided at https://complimet.ca/sidco. Supplementary Material shows an example of an application of SiDCo in metabolomics.

## 1 Introduction

The analysis of biological networks, as a parallel investigation to the study of individual feature characteristics, requires robust quantification of the interconnections between features within biological systems (Ma'ayan 2011). Several methods for data-driven network determination of feature interconnections have been used in the analysis of metabolomic data. Pearson or Spearman correlation-based methods are arguably the most prevalent (Amara et al. 2022). While providing critical information about the direction of dependencies, both methods measure linear or monotonic correlations and cannot detect non-linear feature interactions (Rosato et al. 2018). Distance correlation, a non-parametric approach for correlation analysis, can measure various types of data relationships (linear and non-linear) as well as the correlations between vectors of different lengths (Gábor and Maria 2009; Székely and Rizzo 2013; Edelmann et al. 2021). In metabolomic and lipidomic datasets, distance correlation can take into consideration the sparse coverage of feature data, the potential for determining non-linear relationships, and the possibly random network topologies associated with metabolism and inherent to lipidomic and metabolomic datasets with zero correlation only obtained for fully independent features.

Despite these advantages, few publications have used distance correlation to analyze metabolomic data (Oliveira et al. 2015; Tang et al. 2019; Cuperlovic-Culf et al. 2021). We suggest that this is, in part, due to the lack of easily accessible implementations. Moreover, no parallel implementation, to our knowledge, allows users to assess partial correlations, calculated as the measure of association between pairs of features while removing the confounding effects of other variables. To address this need and thereby provide new methods for the reconstruction of regulatory metabolomic and lipidomic networks, we present here SIgned Distance COrrelation (SiDCo), a web-based application of both signed distance correlation and partial distance correlation implemented using the Gaussian Graphical Model (GGM) previously implemented for other correlation approaches (Lauritzen 1996).

## 2 Implementation

SiDCo is implemented in Python with a RShiny front-end. It is compatible with all web browsers. Two analytical tabs allow users to perform either distance correlation or partial distance correlation. In both applications, users define their desired threshold values and *P* values. Data are automatically z-score normalized across all samples prior to analysis. Users are reminded that missing values must be imputed according to their specifications or data will be returned with the descriptive error message.

Distance correlations and *P* values are calculated and presented as described below and a correlation directionality sign is derived from Pearson correlation analysis as an indication of the overall linear trend in the data. Distance correlation calculations in SiDCo are provided in three forms: (i) “one-to-one,” calculating correlations between each pair of features, (ii) “one-to-all,” providing correlations for each feature with all other features combined, and (iii) partial correlation calculated for each pair of features while controlling for the contributions of other features, i.e. covariates.

Distance correlation, $\mathrm{dCor}\left(X,Y\right)$ between features *X* and *Y* and distance covariance, $\mathrm{dCov}\left(X,Y\right)\;$are calculated as: where *A* and *B* represent doubly centered distance matrices for variables *X* and *Y*, respectively, measured in *n* samples. Distance variances (*dVar*) are: $\mathrm{dVar}\left(X\right)=\frac{1}{n^{2}}\sum_{j=1}^{n}\sum_{k=1}^{n}{A_{j,k}}^{2}$ and $\mathrm{dVar}\left(Y\right)=\frac{1}{n^{2}}\sum_{j=1}^{n}\sum_{k=1}^{n}{B_{j,k}}^{2}$


$$\mathrm{dCor}\left(X,Y\right)=\frac{\mathrm{dCov}\left(X,Y\right)}{\sqrt{d\mathrm{Var}\left(X\right)d\mathrm{Var}\left(Y\right)}},\;{\mathrm{dCov}\left(X,Y\right)}^{2}=\frac{1}{n^{2}}\sum_{j=1}^{n}\sum_{k=1}^{n}A_{j,k}B_{j,k},$$

In a one-to-one correlation calculation, an array of values for each feature is compared with an array of values for all the other features one at a time. In this case, a doubly centered distance matrix is calculated as:

$A_{j,k}=a_{j,k}-{\overset{-}{a}}_{j.}-{\overset{-}{a}}_{k.}+{\overset{̿}{a}}_{.}$ and $B_{j,k}=b_{j,k}-{\overset{-}{b}}_{j.}-{\overset{-}{b}}_{k.}+{\overset{̿}{b}}_{.}$; where Euclidean distance is used to calculate $x_{j}$ to $x_{k}$ or $y_{j}$ to $y_{k}\;as\;$.$a_{j,k}=\sqrt{(x_{j}-x_{k})(x_{j}-x_{k})\prime }$ and $b_{j,k}=\sqrt{(y_{j}-y_{k})(y_{j}-y_{k})\prime }$. ${\overset{-}{a}}_{j.}$, ${\overset{-}{b}}_{j.}$and ${\overset{-}{a}}_{k.}\;{\overset{-}{b}}_{k.}\;$are the *j*-row and *k*-column mean values and ${\overset{̿}{a}}_{.}$, ${\overset{̿}{b}}_{.}\;$are the overall mean of matrices *A* and *B*.

In a one-to-all case, the distance covariance for each feature out of *m* features in *n* dimensional sample space is compared to that of all the other features in *n* x (*m*-1) dimensional space. The doubly centered distance matrix for variable *Y* used in the calculation of *dCov* is here: $b_{j,k}=\sqrt{\sum_{s=1}^{m-1}\left(y_{js}-y_{ks}\right){\left(y_{js}-y_{ks}\right)}^{\prime }}$ and equivalent for $a_{j,k}$ for *X*.

The distance correlation *P* value is calculated using the Student’s *t* cumulative distribution function with *t* value calculated as:

$t(X,Y)=\mathrm{dCor}(X,Y)\;\sqrt{n-2}/\sqrt{1-{\mathrm{dCor}(X,Y)}^{2}}$ and corresponding two-sided *P* value for the *t*-distribution with *n*-2 degrees of freedom. The sign of the distance correlation is given by the sign of the Pearson correlation following (Pardo-Diaz et al. 2021). The final output is provided as an .xlsx file and includes distance correlation and corresponding *P* values. Output of one-to-one analysis also includes the Pearson and Spearman correlations and their corresponding *P* values for completeness.

Partial correlation, the correlation between two features corrected for contributions of other features, is calculated as (following GGM):


$$\rho_{i,j\;.\;\forall k\neq i,j}=-\frac{\omega_{ij}}{\sqrt{\omega_{ii}\omega_{jj}}}.$$

Where matrix $\omega =\Sigma^{-1}$ is inverse of Σ - distance covariance matrix. The inverse of the distance covariance matrix uses the Moore–Penrose method for pseudo-inverse which is equivalent to standard inversion for non-singular square matrices and multiplicative inverse for singular matrices where inverse is not possible. Partial distance correlation calculation should only be performed when number of features is smaller than number of samples. Here *P* values are calculated using the Fisher z-transformed correlation values: $z_{ij}=0.5*\log\frac{1+\rho_{ij}}{1-\rho_{ij}}$ and cumulative standard normal distribution (*cdtf*) function:$\;p_{i,j}=2(1-\mathrm{cdtf}(z_{ij}*\sqrt{N-M-1})$). Results are provided as .xlsx downloads.

## 3 Conclusion

SiDCo is an open-access Web-based application for the calculation of signed and partial distance correlations between features available at https://complimet.ca/SiDCo where detailed instructions are provided.

## Supplementary Material

btad210_Supplementary_Data

## References

[btad210-B1] Amara A , Frainay C, Jourdan F et al Networks and graphs discovery in metabolomics data analysis and interpretation. Front Mol Biosci 2022;9:841373. 10.3389/fmolb.2022.841373. 35350714 PMC8957799

[btad210-B2] Cuperlovic-Culf M , Cunningham EL, Teimoorinia H et al Metabolomics and computational analysis of the role of monoamine oxidase activity in delirium and SARS-COV-2 infection. Sci Rep 2021;11:10629. 10.1038/s41598-021-90243-1. 34017039 PMC8138024

[btad210-B3] Edelmann D , Móri TF, Székely GJ. On relationships between the Pearson and the distance correlation coefficients. Stat Probab Lett 2021;169:108960. 10.1016/j.spl.2020.108960.

[btad210-B4] Gábor JS , Maria LR. Brownian distance covariance. Ann Appl Stat 2009;3:1236–65. 10.1214/09-AOAS312F. PMC2889501 20574547

[btad210-B5] Lauritzen SL. Graphical Models. Oxford: Clarendon Press, 1996.

[btad210-B6] Ma'ayan A. Introduction to network analysis in systems biology. Sci Signal 2011;4:tr5. 10.1126/scisignal.2001965. 21917719 PMC3196357

[btad210-B7] Oliveira AP , Dimopoulos S, Busetto AG et al Inferring causal metabolic signals that regulate the dynamic TORC1-dependent transcriptome. Mol Syst Biol 2015;11:802. 10.15252/msb.20145475. 25888284 PMC4422559

[btad210-B8] Pardo-Diaz J , Bozhilova LV, Beguerisse-Díaz M et al Robust gene coexpression networks using signed distance correlation. Bioinformatics 2021;37:1982–9. 10.1093/bioinformatics/btab041. 33523234 PMC8557847

[btad210-B9] Rosato A , Tenori L, Cascante M et al From correlation to causation: analysis of metabolomics data using systems biology approaches. Metabolomics 2018;14:37. 10.1007/s11306-018-1335-y. 29503602 PMC5829120

[btad210-B10] Székely GJ , Rizzo ML. Partial distance correlation with methods for dissimilarities. *arXiv: Methodology* 2013;1310.2926.

[btad210-B11] Tang ZZ , Chen G, Hong Q et al Multi-omic analysis of the microbiome and metabolome in healthy subjects reveals microbiome-dependent relationships between diet and metabolites. Front Genet 2019;10:454. 10.3389/fgene.2019.00454. 31164901 PMC6534069

