# Supplementary material for: Signed Distance Correlation (SiDCo): an online implementation of distance correlation and partial distance correlation for data-driven network analysis
Source: Bioinformatics. 2023 May 3;39(5):btad210. doi: 10.1093/bioinformatics/btad210 (PMC10353719; doi:10.1093/bioinformatics/btad210)
Supplement: btad210_Supplementary_Data [file btad210_supplementary_data.docx]

**Signed Distance Correlation (SiDCo): an online implementation of distance correlation and partial distance correlation for data-driven network analysis**

Francesco Monti^1,2+^, David Stewart^1,2+^, Anuradha Surendra^1,2^, Irina Alecu^2,3^, Thao Nguyen-Tran^2-4^, Steffany A.L. Bennett^2-4*^, Miroslava Čuperlović-Culf^1-3^**^*^**

^1^National Research Council of Canada, Digital Technologies Research Centre, Ottawa, Ontario, Canada, ^2^Neural Regeneration Laboratory and India Taylor Lipidomic Research Platform, Ottawa, Ontario, Canada, ^3^Department of Biochemistry, Microbiology, and Immunology and Ottawa Institute of Systems Biology, Ottawa, Ontario, Canada, ^4^Department of Chemistry and Biomolecular Sciences, Centre for Catalysis Research and Innovation, University of Ottawa, Ottawa, Ontario, Canada

+ Equal first authors

*To whom correspondence should be addressed.

**Supplementary material**

We present here several possible use-cases and visualization possibilities of the analyses provided by SiDCo. Using the publicly available metabolomic dataset of Li et al. (1), we randomly selected 50 metabolite profiles for 413 cancer cell lines. Presented visual representations of SiDCo results are performed using in house Matlab code (Matworks Inc) and Cytoscape (2).

| Figure S1 | Comparison of distance correlation with p-value <0.01 and partial distance correlation with p-value <0.01. |
| --- | --- |
| Figure S2 | Principal Component Analysis (PCA) of distance correlation matrix showing A. groupings of metabolites based on edge values; B. Biplot of the significance of metabolites in major variances projections as an alternative way to show groupings of distance correlation nodes. |
| Figure S3 | Partial distance correlation values with p<0.01 and correlation >0.2 shown as a metabolic network. |

Example datasets and detailed instructions for SiDCo are provided at: <https://complimet.ca/sidco/>.

**Figure S1.** Comparison of correlation values for the Li et al (1) metabolomic dataset calculated using signed distance correlation (upper right triangle) and partial distance correlations (lower left triangle). Shown are values in both cases with p<0.01 and no correlation thresholding. Removal of indirect, i.e., correlation through a third partner provided by partial distance correlation leads to a significantly less dense network.


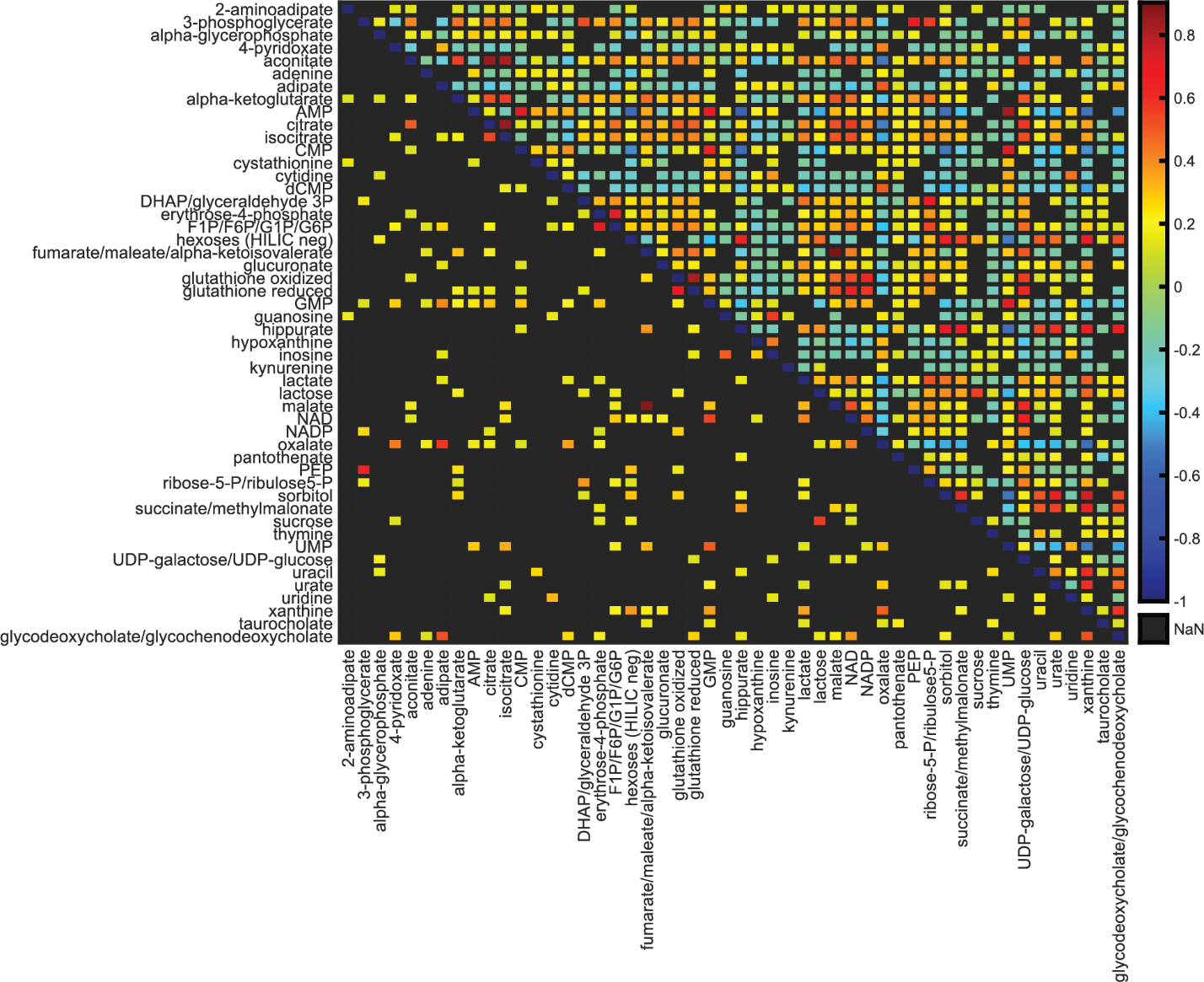


**Figure S2.** Principal Component Analysis (PCA) of feature correlations across all cell lines. This analysis shows the grouping of features, i.e., potential nodes of a distance correlation network. PCA representation of this result can be provided either through plotting of principal components and viewing features as points on the graph by plotting the rows of the distance correlation matrix generated by SidCo (A). Alternatively, the contribution of each feature, i.e., plotting the column inthe distance correlation matrix generated by SidCo, can be presented as a PCA bi-plot (B). Both approaches show groups of nodes that represent distance correlation networks.


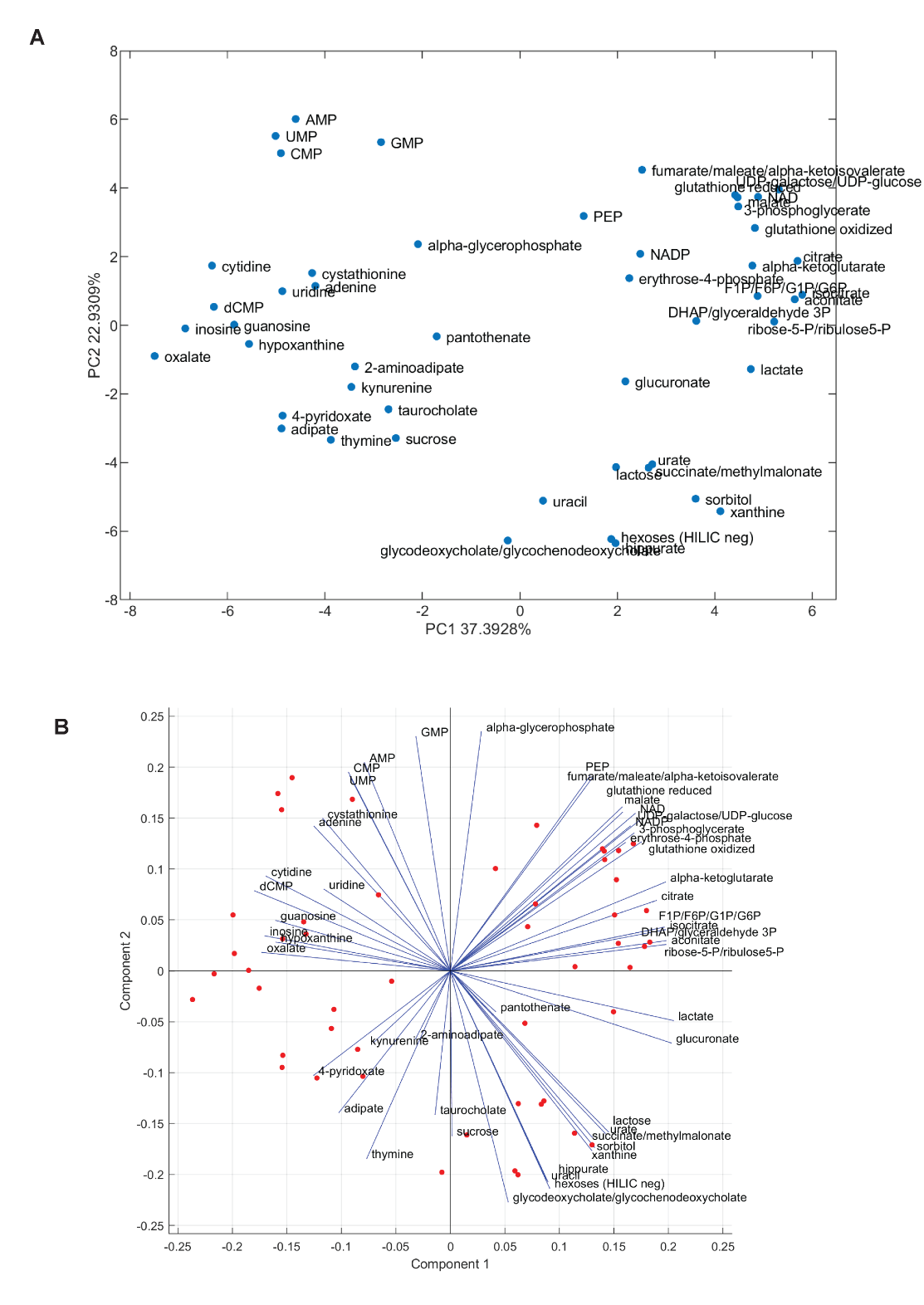


**Figure S3.** Removal of indirect correlation provided through partial distance correlation analysis leads to a significantly less dense network that can be graphically represented as a metabolic network. Here we include as non-zero edges between metabolites features with partial distance correlation values of p<0.01 and correlation values higher then 0.2 representing the user-defined thresholds entered into SiDCo. Network representation was generated using Cytoscape (2).


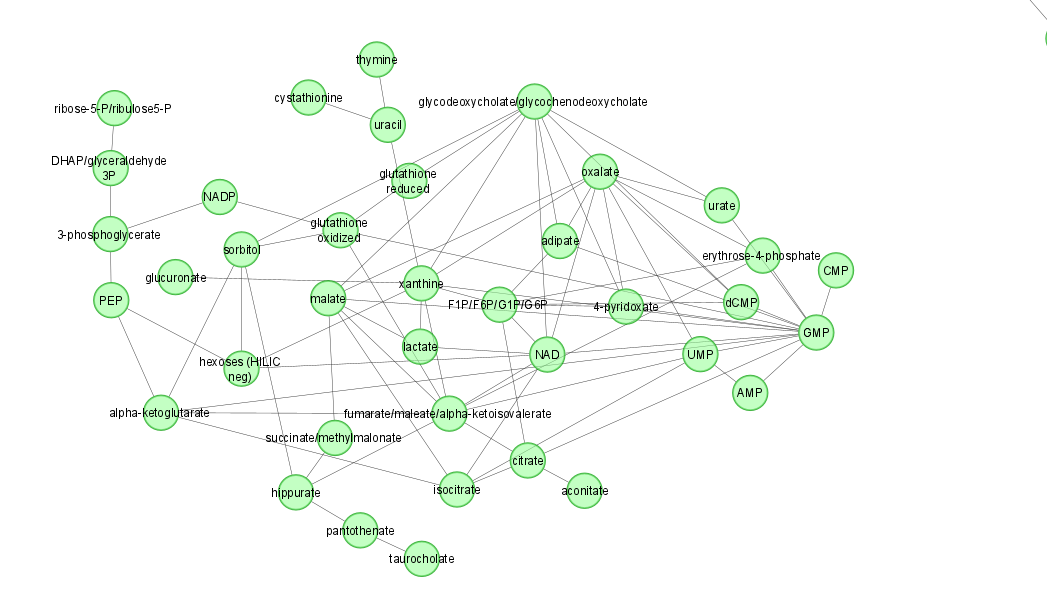


**References**

1. Li, H., Ning, S., Ghandi, M. et al. The landscape of cancer cell line metabolism. Nat Med 25, 850–860 (2019).
2. Shannon P, Markiel A, Ozier O, Baliga NS, Wang JT, Ramage D, Amin N, Schwikowski B, Ideker T. Cytoscape: a software environment for integrated models of biomolecular interaction networks. Genome Research, 13:2498-504 (2003) https://cytoscape.org/
